# Machine Learning Algorithms for Risk Prediction of Severe Hand-Foot-Mouth Disease in Children

**DOI:** 10.1038/s41598-017-05505-8

**Published:** 2017-07-14

**Authors:** Bin Zhang, Xiang Wan, Fu-sheng Ouyang, Yu-hao Dong, De-hui Luo, Jing Liu, Long Liang, Wen-bo Chen, Xiao-ning Luo, Xiao-kai Mo, Lu Zhang, Wen-hui Huang, Shu-fang Pei, Bao-liang Guo, Chang-hong Liang, Zhou-yang Lian, Shui-xing Zhang

**Affiliations:** 1 Department of Radiology, Guangdong General Hospital/Guangdong Academy of Medical Sciences, Guangzhou, Guangdong P.R. China; 2 Graduate College, Southern Medical University, Guangzhou, Guangdong P.R. China; 3 Institute of Computational and Theoretical Study and Department of Computer Science, Hong Kong Baptist University, Hong Kong, P.R. China; 4 Department of Radiology, The First People’s Hospital of Shunde, Foshan, Guangdong P.R. China; 5 Department of Mathematics, Hong Kong Baptist University, Hong Kong, P.R. China; 6 Department of Radiology, Huizhou Municipal Central Hospital, Huizhou, Guangdong P.R. China

## Abstract

The identification of indicators for severe HFMD is critical for early prevention and control of the disease. With this goal in mind, 185 severe and 345 mild HFMD cases were assessed. Patient demographics, clinical features, MRI findings, and laboratory test results were collected. Gradient boosting tree (GBT) was then used to determine the relative importance (RI) and interaction effects of the variables. Results indicated that elevated white blood cell (WBC) count > 15 × 10^9^/L (RI: 4^9^.47, p < 0.001) was the top predictor of severe HFMD, followed by spinal cord involvement (RI: 26.62, p < 0.001), spinal nerve roots involvement (RI: 10.34, p < 0.001), hyperglycemia (RI: 3.40, p < 0.001), and brain or spinal meninges involvement (RI: 2.45, p = 0.003). Interactions between elevated WBC count and hyperglycemia (H statistic: 0.231, 95% CI: 0–0.262, p = 0.031), between spinal cord involvement and duration of fever ≥3 days (H statistic: 0.291, 95% CI: 0.035–0.326, p = 0.035), and between brainstem involvement and body temperature (H statistic: 0.313, 95% CI: 0–0.273, p = 0.017) were observed. Therefore, GBT is capable to identify the predictors for severe HFMD and their interaction effects, outperforming conventional regression methods.

## Introduction

Hand-foot-mouth disease (HFMD) is a common infectious disease caused by a group of enteroviruses, with enterovirus 71 (EV-71) and Coxsackie virus A16 (CA-V16) being the most prevalent in China^1, 2^. Over the last decade, outbreaks of HFMD that were associated with EV71 have been reported in countries in the Western Pacific Region, including Japan, Malaysia, Singapore, and China^3, 4^. The cumulative total of the reported cases in China has reached approximately 1.7 million, 1.9 million, and 2.7 million in 2010, 2013, and 2014, respectively^5, 6^. The clinical manifestations of most HFMD cases were mild and limited to fever, rash, or herpes on hand, foot, and mouth^7^. In general, mild infections are self-limited and not life-threatening, while severe HFMD are often associated with neurological and systemic complications, such as aseptic meningitis, brainstem encephalitis, acute flaccid paralysis, myocarditis and pulmonary oedema that requires hospitalization, or even causing death^2, 8^. Unfortunately, the incidence of severe HFMD in mainland China is high.

Evidences from global reports on HFMD epidemics have substantiated that the incidence of severe HFMD is elevating gradually, along with mortality rate^9^. Thus, identifying potential early indicators for severe HFMD is essential, which enable early medical interventions and alleviating the disease severity, subsequently reducing the mortality rate. Previous studies have found that the following conditions or practices were associated with the increased risk of severe HFMD: a duration of fever ≥3 days, body temperature ≥37.5 °C, fatigue, the use of glucocorticoids, the use of dehydrant drugs, maculopapular rash, hyperglycemia, vomiting, EV71 infection, attending home care, neutrophilia, and young age^10–18^. Although specific clinical manifestations have been identified using the Magnetic Resonance Imaging (MRI), the potential indicators of the disease severity were not validated. Furthermore, the relative importance of each MRI-related factor and interaction effects of the clinical indicators still remain unclear.

Hence, the aim of this study was to identify clinical and MRI-related predictors for the occurrence of severe HFMD in children and to assess the interaction effects between them using machine learning algorithms.

## Methods

### Ethics statement

This retrospective study has been approved by the Guangdong General Hospital review board, in which the informed consent was not required from the patients. All experiments were performed in accordance with the relevant guidelines and regulations. Patients identifiers were anonymised to protect the privacy of the patients.

### Clinical criteria

The following clinical symptoms were used to detect HFMD in children: maculopapular or vesicular rash on the palms and/or soles, and vesicles or ulcers in the mouth. The diagnoses were confirmed by the isolation of enteroviruses such as EV71 and CA16, from at least one type of sample (throat swab, blood, stool, cerebrospinal fluid, or other). Mild HFMD was defined as vesicular skin rash on hand, foot, mouth, or buttock. Severe HFMD was similar to mild HFMD with the addition of neurological, cardiorespiratory complications that could lead to death. Neurological complications included aseptic meningitis, encephalitis, and acute flaccid paralysis, while cardiorespiratory complications were characterized by the presence of respiratory distress, tachycardia, pulmonary oedema, and pulmonary congestion.

Hypertension was defined as blood pressure of >20 mmHg. Blood pressure was calculated as follows: systolic blood pressure (SBP) = (age × 2) + 80 mmHg; diastolic blood pressure (DBP) = 2/3 SBP; and blood pressure will be SBP/DBP mmHg. The duration of fever was defined as the duration (days) of body temperature ≥37.5 °C; elevated WBC count was defined as peripheral WBC ≥ 15 × 10^9^/L; hyperglycemia was defined as blood glucose concentration of >8.3 mmol/L during admission; tachycardia was defined as heart rate of over 160 beats/min, 140 beats/min, 120 beats/min for infant, toddler, and child, respectively; and muscle strength was graded using the Lovett method (grades 0-V), while the muscle weakness was graded from 0-IV.

### Patient recruitment and treatment

Guangdong General Hospital is a 2852-bed tertiary teaching hospital in Guangdong province, providing services to about 5.6 million patients per year. Most patients reside in rural and regional centres or townships. The hospital is one of the designated referral centres for HFMD in the region, and all identified HFMD patients are required to be hospitalized and monitored in the paediatrics unit. A retrospective review of HFMD patients who were admitted to the paediatric unit from January 2009 to December 2014 was performed. The Chinese guideline for HFMD diagnosis and treatment (Chinese Ministry of Public Health, revised in 2010) was used as reference for clinical diagnosis of HFMD, and only those patients who were newly diagnosed with HFMD and not treated were included in this study. Patients were categorized into the following groups based on the severity of the infection: (1) mild HFMD without severe complications; (2) severe HFMD with severe complications.

Before the treatment, brain and spinal MRI was taken regularly for all the HFMD patients at the early stage of infection (1–4 days after the onset of symptoms) except for those relatively milder HFMD cases, in which the patients were exempted from undergoing MRI. Assays for the detection of EV71 infection and determination of WBC count were also performed. For mild HFMD, they were treated with paracetamol and sufficient water, while for severe HFMD, glucocorticoid, oxygen, anti-virus drugs and/or intravenous immunoglobulin were administered.

### Data collection

Demographic characteristics, clinical symptoms and signs, EV-A71 test results, WBC count, chest radiograph, and MRI reports of the patients were collected.

Demographics include age and sex; clinical symptoms and signs include oral ulcers, rash or herpes, duration of fever, vomiting, tachycardia, convulsion, altered consciousness including irritability, lethargy, drowsiness, and/or coma, neck stiffness or positive Kerning’s sign, muscle weakness, breathlessness, hypertension and elevated body temperature.

### MRI protocol

1.5T MRI (GyroscanAchieva 1.5 T, Philips Healthcare, Best, Netherlands) equipped with an 8-channel head coil was used to performed the scan. The acquisition parameters were as follows: pre-contrast T1-weighted (T1-w) images (TR/TE = 593/15 ms, FOV = 18 × 18 cm, matrix = 256 × 256, slice thickness = 5 mm, spacing = 1.0 mm); T2-weighted (T2-w) images (TR/TE = 3720/100 ms, FOV = 18 × 18 cm, matrix = 256 × 256, slice thickness = 5 mm, spacing = 1.0 mm); T2/fluid-attenuated inversion-recovery (T2/FLAIR) images (TR/TE: 11000/140 ms, range of inversion time = 2400 ms, FOV = 18 × 18 cm, matrix = 256 × 256, slice thickness = 5 mm, spacing = 1.0 mm); and contrast-enhanced T1-w images (TR/TE: 488/15 ms, FOV = 18 × 18 cm, matrix = 256 × 256, slice thickness = 5 mm, spacing = 1.0 mm). Contrast MRI was performed using GD-DTPA (Bayer, Germany) at a dose of 0.1 mmol/kg.

### Imaging analysis

All MRI examinations were reviewed independently by two neuroradiologists with 20 years of experience. Locations of the lesion were analysed on T1-w, T2-w, and T2/FLAIR as follows: brain or spinal meninges, cerebrum and cerebellum (cerebral cortex, cerebral white substance basal ganglia, callosum, thalamus, and cerebellum), brainstem (midbrain, pons and medulla oblongata), spinal cord (cervical, thoracic and lumbar segments), and spinal nerve roots. The lesions were defined as either hypointense on T1-w, hyperintense on T2-w and T2/FLAIR images, or enhanced on T1-w contrast images.

### Statistical analysis

In this study, gradient boosting tree (GBT) was chosen to assess the interaction effects due to the following rationale: (1) Gradient boosting is a powerful machine learning technique combining the algorithms of decision trees and boosting, which can handle complex interaction effects that conventional approaches lack; (2) GBT can handle different types of predictor variables and missing data by boosting, using only the complete predictors; (3) Elimination of outliers and prior data transformation are not required; (4) With the non-linear GBT formulation, a robust non-linear interactions can be provided. Therefore, GBT is suitable for handling the selected interaction effects in this study^19^.

For single variable, the relative importance (RI) was calculated using the number of times a variable was selected for splitting, weighted by the squared improvement to the model as a result of each split, and averaged over all trees. The relative importance of all variables were normalized and scaled to have a maximum value of 100. For the interaction, Friedman’s H-statistic was used to assess the relative strength of interaction effects in non-linear models with a scale of 0 to 1, with higher values indicating stronger interaction effects.

The prediction performance of GBT is based on the average results over all 10 testing data sets. However, to find the interactions among factors and also conduct the statistical inference on those interactions, we need to decide which configuration (the number of trees) should be used for the permutation test. In our study, we chose the setting based on the 10 fold validation. For the prediction performance of GBT, we compared the results of using the down-sampling strategy to balance our training-test data with that of no using any balance approach.

A permutation test is a type of statistical significance test, in which the distribution of the test statistic under the null hypothesis is obtained by calculating all possible values of the test statistic under rearrangements of the values on the observed data points. The ranking of the real test statistic among the shuffled test statistics gives the p-value. The standard p-value threshold of 0.05 was then used to select attributes and interactions.

Statistical analysis was performed with the R software (R Core Team. R: A language and environment for statistical computing. R Foundation for Statistical Computing, Vienna, Austria. http://www.R-project.org, 2016). The packages in R could be found in: https://cran.r-project.org/web/packages/gbm/gbm.pdf, including ‘gbm’, ‘glmnet’, ‘caret’, ‘gtools’, ‘ggplot2’, ‘gplots’, and ‘ROCR’. A p-value of <0.05 was deemed to be statistically significant in two-sided tests.

## Results

### Patient characteristics

A total of 1172 patients infected with HFMD were assessed, in which laboratory results were not available for 83 patients; pre-treatment MRI scan was not performed in 347 relatively milder patients; and the remaining 212 patients had received treatment in other hospitals. Finally, a total of 530 patients were recruited in this study. The comparison of the demographic and clinical data between 185 severe HFMD and 345 mild HFMD patients are shown in Table 1. Complications detected in the severe HFMD patients were as follows: aseptic meningitis (n = 13), encephalitis (n = 99), and acute flaccid paralysis (n = 59), cardiorespiratory complications (n = 103), and death (n = 0).

**Table 1:** Comparison of demographic and clinical data between mild HFMD and severe HFMD patients.

Characteristics*	Mild HFMD (n = 345)	Severe HFMD (n = 185)	p-value
Predictive variables			
Age, months (mean ± SD)	27.1 ± 17.8	28.4** ± **22.9	0.447
Male	245 (71.0%)	134 (72.4%)	0.172
Body temperature (mean ± SD)	38.96** ± **0.64	38.98 ± 0.62	0.746
Duration of fever ≥3 days	60 (17.4%)	106 (57.3%)	<0.001
WBC count >15 × 10^9^/L	29 (8.4%)	126 (68.1%)	<0.001
Hypertension	17 (4.9%)	22 (11.9%)	0.003
Hyperglycemia	51 (14.8%)	82 (44.3%)	<0.001
Rash or herpes	333 (96.5%)	180 (97.3%)	0.629
EV71-positive	284 (82.3%)	111 (60.0%)	<0.001
Outcome variables			
Limb weakness	0 (0%)	59 (31.9%)	<0.001
Tachycardia	0 (0%)	10 (5.4%)	<0.001
Muscle weakness	0 (0%)	38 (20.5%)	<0.001
Irritating vomiting	0 (0%)	30 (16.2%)	<0.001
Breathlessness	0 (0%)	13 (6.9%)	<0.001
Altered consciousness	0 (0%)	99 (53.5%)	<0.001
Positive Kerning’s sign	0 (0%)	13 (7.0%)	<0.001
Convulsion	0 (0%)	70 (37.8%)	<0.001
Pulmonary oedema	0 (0%)	10 (5.4%)	<0.001

HFMD = Hand-Foot-Mouth Disease; *Except where otherwise indicated, values are the number of patients (percentage) of patients with the characteristic.

### MRI findings

The comparison of MRI findings between mild and severe HFMD in patients was shown on Table 2. The results have demonstrated that positive imaging findings were more likely being detected in severe HFMD patients than in mild HFMD patients (p < 0.01).

**Table 2:** Comparison of MRI findings between mild HFMD and severe HFMD.

Involved areas	Mild HFMD (n = 345)	Severe HFMD (n = 185)	p-value
Cerebrum and cerebellum	8 (2.3%)	14 (7.6%)	0.004
Brain or spinal meninges	16 (4.6%)	20 (10.8%)	0.007
Brainstem	49 (14.2%)	44 (23.8%)	0.006
Spinal cord	0 (0.0%)	60 (32.4%)	**<**0.001
Spinal nerve roots	26 (7.5%)	28 (15.1%)	0.010

Note: HFMD = Hand-Foot-Mouth Disease; categorical variables were expressed as number of patients (percentage); Cerebrum and cerebellum include cerebral cortex, cerebral white matter, cerebellum, basal ganglia, callosum and thalamus; Brainstem includes medulla oblongata, pons, and midbrain. Spinal cord includes cervical cord, thoracic cord, and lumbar spinal cord.

### Predictors for severe HFMD patients

**Figure 1:**
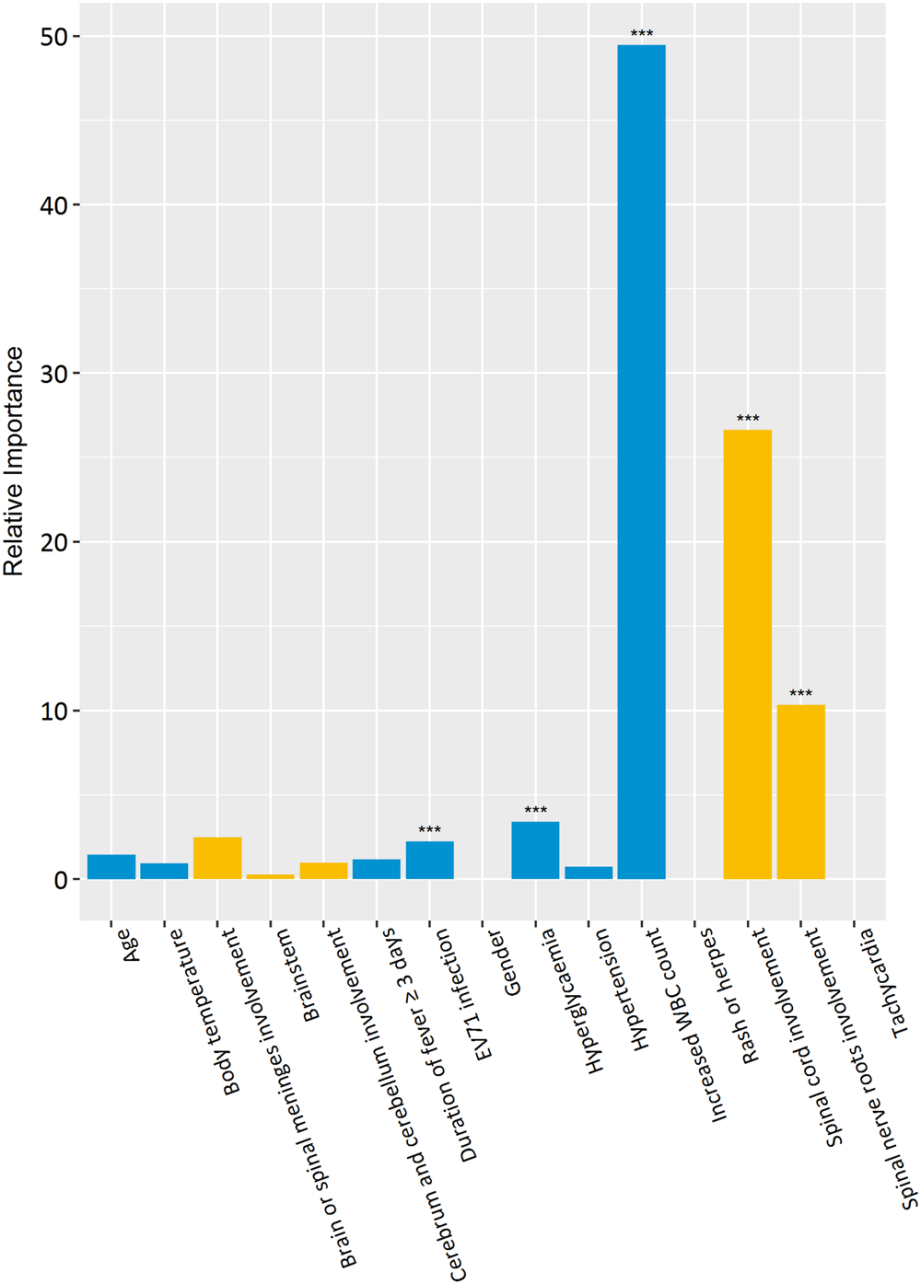
Relative importance (RI) for each individual predictor. Note that the *indicated the level of significance, p-value < 0.001. MRI-related predictors were in yellow and clinical predictors were in blue.

The gradient boosting tree approach was used to determine the relative importance of a single variable. In the multivariate analysis, an elevated WBC count ≥15 × 10^9^/L (RI: 49.47, p < 0.001) was shown to be the top indicator for severe HFMD, followed by spinal cord involvement (RI: 26.62, p < 0.001), spinal nerve roots involvement (RI: 10.34, p < 0.001), hyperglycemia (RI: 3.40, p < 0.001), brain or spinal meninges involvement (RI: 2.45, p = 0.003), and EV-A71 infection (RI: 2.24, p < 0.001) (Fig. 1). Only a very small or zero relative importance was found in age (RI: 1.44, p = 0.778), cerebrum and cerebellum involvement (RI: 0.95, p = 0.03), body temperature (RI: 0.93, p = 0.854), hypertension (RI: 0.72, p = 0.077), brainstem involvement (RI: 0.27, p = 0.316), gender (RI: 0.00, p = 0.916), rash or herpes (RI: 0.00, p = 0.018), and tachycardia (RI: 0.00, p = 0.018) (Fig. 1). All p-values were calculated using the permutation test, described in the statistical analysis section (Supplementary Figure 1).

### The predictive performance of GBT-based model

If we didn’t use any balance approach, the performance of GBT model can achieve 92.3% prediction accuracy. The area under the receiver operating characteristic curve (AUC) is 0.985, with a sensibility of 0.85, a specificity of 0.97 (Supplementary Figure 2). After balancing the training-test data using a down-sampling procedure, the prediction accuracy of GBT is a slightly worse than 92.3%, which is 89.2%. The AUC of model is 0.948, with a sensibility of 0.80, a specificity of 0.93 (Supplementary Figure 3).

### Interaction effects between predictors

**Figure 2:**
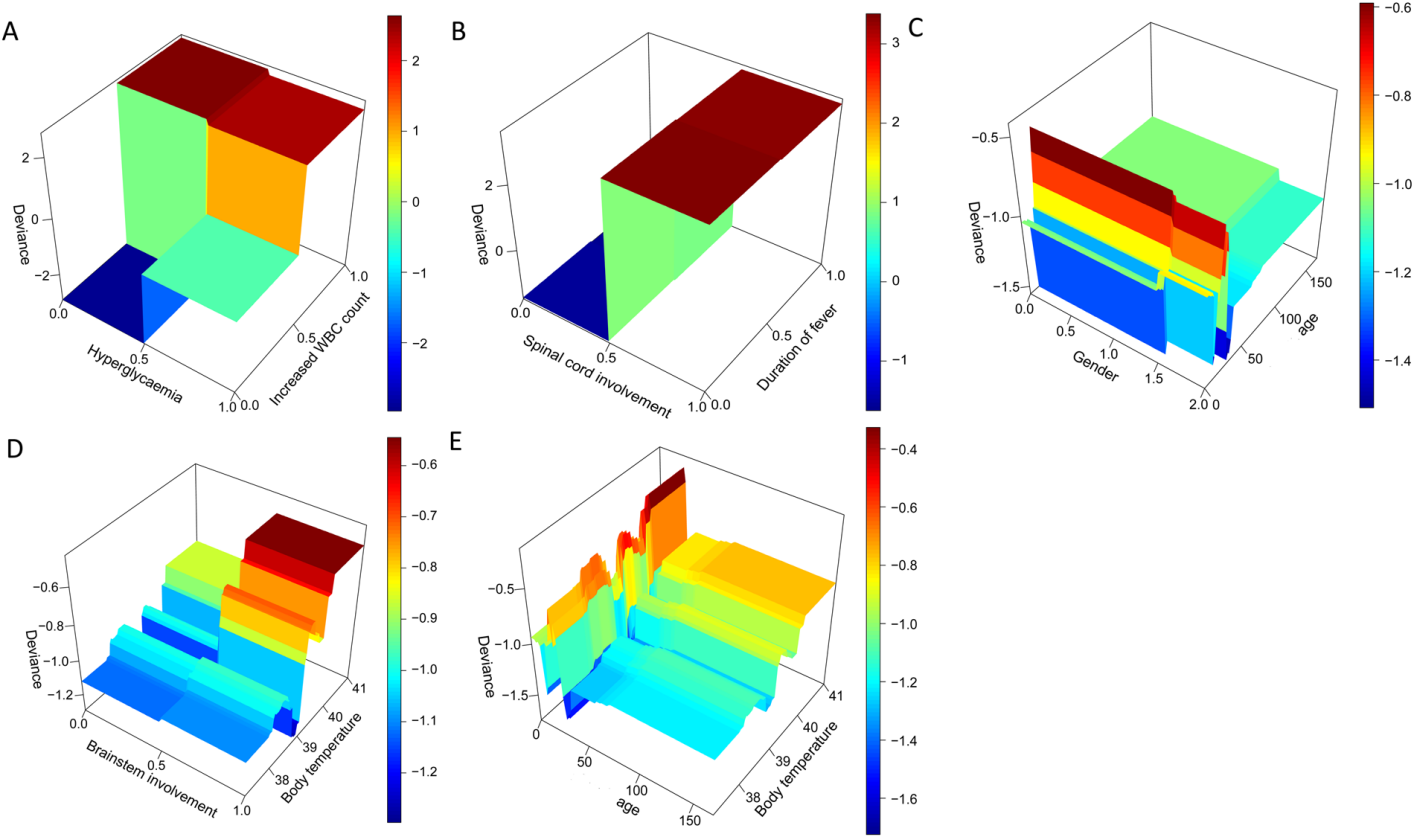
The interaction effect between two predictors. The x-axis denotes the status of the predictor. The y-axis (deviance) is a quality-of-fit statistic for the interaction that measures how well the new configuration fits the data. (In Fig. 2A, we can observe a significant increase in the risk of severe HFMD when the probability of increased WBC count and hyperglycemia changed from 0.5 to 1. In Fig. 2B, we can observe that conditioning on the change of spinal cord involvement, the effect of duration of fever ≥3 days is minor. In Fig. 2C, when the age is between 0 and 50 months and the patient is male, we can observe a significant increase risk of severe HFMD. In Fig. 2D, we can observe that if we fix the body temperature at 38 degree, then there is a slight increase in the risk of severe HFMD when the probability of brainstem involvement changed from 0 to 1. However, when the body temperature is between 40 and 41 degree, we can observe a significant increase in the risk of severe HFMD if the status of brainstem involvement changed. In Fig. 2E, when the body temperature is between 40 and 41 degree and the age is between 0 and 50 months, we can observe a significant increase risk of severe HFMD.

Interaction between the two exposures was defined as the effect of one exposure on an outcome, depending on the presence or absence of another exposure. The stronger the dependence between these two exposures, the stronger the interaction between them was. To interpret the interaction between the two exposures, one exposure was fixed at constant while others changed, and the risk of severe HFMD was assessed. Three pairs of interactions were found to be statistical different in the present study. Figure 2 illustrated the interaction between elevated WBC count and hyperglycemia (H statistic: 0.231, 95% CI: 0–0.262, p = 0.031), between spinal cord involvement and duration of fever (H statistic: 0.291, 95% CI: 0.035–0.326, p = 0.035), and between brainstem involvement and body temperature (H statistic: 0.313, 95% CI: 0–0.273, p = 0.017). The H statistic for interaction between age and body temperature was 0.244 (95% CI: 0.05–0.424, p = 0.251), while the interaction between age and gender was 0.168 (95% CI: 0–0.275, p = 0.092). A significant increase in the risk of severe HFMD was observed when the probability of the elevated WBC count and hyperglycemia increased from 0.5 to 1 (Fig. 2A). We can observe that conditioning on the change of spinal cord involvement, the effect of duration of fever is minor (Fig. 2B). A significant increase risk of severe HFMD was observed in male patients aged 0 to 50 months (Fig. 2C). When the body temperature was constant (38 °C), there was a slight increase in the risk of severe HFMD when the probability of brainstem involvement increased from 0 (no brainstem involvement) to 1 (brainstem involvement). However, when the body temperature was between 40 °C and 41 °C, a significant increase in the risk of severe HFMD was observed if the probability of brainstem involvement changed, or when the age range was 0 to 50 months (Fig. 2E). All p-values were calculated using permutation test (Supplementary Figure 2).

## Discussion

In this study, a powerful machine-learning algorithm, GBT was used to analyse the predictors for severe HFMD in children. Results have shown that elevated WBC count (≥15 × 10^9^/L) as the top indicator associated with the risk of severe HFMD, followed by spinal cord involvement, spinal nerve roots involvement, hyperglycemia and brain or spinal meninges involvement. The GBT model achieved an AUC of 0.985, indicating GBT as a good predictive model. In addition, this is the first study to describe interactions between elevated WBC count and hyperglycemia, between spinal cord involvement and duration of fever, and between brainstem involvement and body temperature.

In China, HFMD has been classified as a category C notifiable infectious disease since May 2, 2008. Although the majority of HFMD episodes are generally mild and self-limiting, the infection may rapidly develop into severe HFMD with serious complications and possibly life-threatening^20, 21^. Therefore, it is necessary to identify predictors for severe HFMD^18^.

Previous studies have shown that EV71 was more likely to cause serious complications than other enteroviruses and usually results in meningoencephalitis, pulmonary haemorrhage, and circulation failure^22, 23^. It is found that EV71 has strong neurotropism, subsequently affects axonal transport in neuron cells, resulting in brain infection and flaccid paralysis^24^. Etiological examination of EV71 infection requires special equipment and is time-consuming, complicating the diagnosis^10^. In this study, higher incidence of EV-A71 infection was found in mild HFMD than in severe HFMD, with low relative importance of EV-A71 infection (RI = 2.24). This may be due to the following reasons: (1) The presence of several outbreaks of HFMD that were associated with EV71 in Guangzhou may have increased the detection of EV-A71 infection in mild HFMD; (2) The exclusion of patients with mild HFMD who were exempted from MRI scan could have contributed to sampling bias. Therefore, having other early predictors for severe HFMD may provide more accurate results for risk prediction.

Although previous studies have identified possible predictors associated with increased probability of severity, such as children who attend child care centres^25^, has leukocytosis^17^, has limb weakness^26^, or has persistent fever^27^, there is still much uncertainty about the relative importance of these predictors. Limited studies have systematically evaluated the criteria for early screening of severe cases and some studies have only categorized severe and mild HFMD based on the length of hospitalization rather than a clinical measure of severity^25^.

This study has shown a comprehensive analysis of mild and severe HFMD compared to previous studies. Besides, this is a novel study that demonstrated clinical manifestations and MRI findings as potential indicators for severe HFMD. Furthermore, the interaction effects of indicators were verified using gradient boosting tree approach.

In this study, mild and severe HFMD were defined based on clinical diagnosis, and the establishment of a gradient boosting tree model, allowing the determination of the relative importance of predictors that are associated with the increased probability of severity. Elevated WBC count was identified as the top predictor of severe HFMD. HFMD patients with elevated WBC count had much higher risk for central nervous system infection^28^. Elevated WBC count was also found to associate with hyperglycemia, another clinical risk factor, which had a relatively small importance of 3.40. Hyperglycemia may result from the loss of blood glucose homeostasis, in which the autonomic nervous system plays an essential part. Upon the stimulation of the sympathetic nervous system, adrenaline and glucagon concentrations increase, while the insulin concentration decreases^20^. Previous studies have shown that young age and male gender were associated with severe HFMD, but there was no evidence showing this in the current study^10, 29^.

Hitherto, several reports have only described the MRI scan characteristics of complications in the central nervous system, resulted from EV71 infection^30–32^. We have identified three MRI-related predictors; they were spinal cord involvement, spinal nerve roots involvement, and brain or spinal meninges involvement. Of these, spinal cord involvement was the most important indicator of severe HFMD, in which the risk was higher accompanied by longer duration of fever. Study has shown that by using immunohistochemistry methods, EV71 antigen could be first detected in the small intestine at 6 hours, in the spinal cord at 24 hours, and in the brainstem at 78 hours^33^. It was also suggested that major pathway for enterovirus entry into the central nervous system is via the peripheral nervous system; subsequently, the enterovirus spreads rostrally up to established neural pathways^34^. Our study may suggest that the EV71 infection pathway initiated from the spinal cord to spinal nerve roots and to the brainstem based on their relative importance. Although the brainstem is the most commonly infected area, the relative importance of brainstem involvement was very small (0.27), which was inconsistent with the findings of other studies^30, 35^. In fact, in our study, most of the HFMD patients with only brainstem involvement had complete recovery, and the lesions on MRI disappeared in two years’ follow-up.

Limitations of this study should also be acknowledged. Firstly, the study was retrospective in nature. Besides, predictors such as plasma cytokine level, the effect of attending home care, living in rural area, and education status were not evaluated because these data were not available. Furthermore, the hospital was used as a reference centre and this may contribute to sample bias. In addition, the presence of selection bias due to the strict criteria used, in which only children who underwent MRI scans were included in the study could also affect the prediction. Lastly, the conventional MRI may not be sensitive enough to detect lesions at the early stage of HFMD, diffusion weighted imaging might be more sensitive in detecting EV71 encephalitis at the early stage.

In conclusion, GBT approach was used to successfully identify the predictors related to severe HFMD and the interaction effects of multiple predictors. With the evidence provided in this study, it is recommended that clinicians should take precaution when children are diagnosed with HFMD, accompanied with elevated WBC count and/or disorders associated with spinal cord involvement, spinal nerve roots involvement, hyperglycemia, and brain or spinal meninges involvement, especially when the first three predictors were detected. It should be noted that the risk of disease severity will significantly increase if the numbers of interacting predictors increase. Although MRI scan was not required for children with mild HFMD, undergoing MRI scan might be useful to rule out the false-negative severe HFMD.

Severe HFMD can be controlled by immediate effective treatment as the early pathological changes are reversible. Early detection and meticulous management of infected HFMD patients are required. Besides, MRI should be used as a routine tool to evaluate the severity and to predict the prognosis of severe HFMD as most of the predictors are MRI-related. In order to reduce the incidence and mortality of severe HFMD, doctors and health care providers will need to be aware of the risk predictors for severe HFMD. Enhanced identification tools for mild HFMD at early stage will be helpful to prevent the progression of mild to severe HFMD.

## Electronic supplementary material

supplementary data

## References

[1] Takahashi S (2016). Hand, Foot, and Mouth Disease in China: Modeling Epidemic Dynamics of Enterovirus Serotypes and Implications for Vaccination. PLoS Med.

[2] Xing W (2014). Hand, foot, and mouth disease in China, 2008-12: an epidemiological study. Lancet Infect Dis.

[3] Yang T (2012). A case-control study of risk factors for severe hand-foot-mouth disease among children in Ningbo, China, 2010–2011. Eur J Pediatr.

[4] Xu W (2012). Distribution of enteroviruses in hospitalized children with hand, foot and mouth disease and relationship between pathogens and nervous system complications. Virol J.

[5] Wang Y (2011). Hand, foot, and mouth disease in China: patterns of spread and transmissibility. Epidemiology.

[6] Liu SL (2015). Comparative epidemiology and virology of fatal and nonfatal cases of hand, foot and mouth disease in mainland China from 2008 to 2014. Rev Med Virol.

[7] Secrest AM, Shah KN (2013). Picture of the month. Hand-foot-and-mouth disease. JAMA Pediatr.

[8] Li W (2014). Study on risk factors for severe hand, foot and mouth disease in China. PLoS One.

[9] Solomon T (2010). Virology, epidemiology, pathogenesis, and control of enterovirus 71. Lancet Infect Dis.

[10] Fang Y (2014). Risk factors of severe hand, foot and mouth disease: a meta-analysis. Scand J Infect Dis.

[11] Zhang D (2017). A Case-control Study on Risk Factors for Severe Hand, Foot and Mouth Disease. Sci Rep.

[12] Long L (2016). Risk factors for death in children with severe hand, foot, and mouth disease in Hunan, China. Infect Dis (Lond).

[13] Chew SP (2015). Risk factors for severe hand foot mouth disease in Singapore: a case control study. Bmc Infect Dis.

[14] Chen SM (2015). Risk Factors for Severe Hand-Foot-Mouth Disease in Children in Hainan, China, 2011-2012. Asia Pac J Public Health.

[15] Song C (2015). Risk factors of severe hand, foot and mouth disease complicated with cardiopulmonary collapse. Infect Dis (Lond).

[16] Owatanapanich S, Wutthanarungsan R, Jaksupa W, Thisyakorn U (2015). Risk Factors for Severe Hand, Foot and Mouth Disease. Southeast Asian J Trop Med Public Health.

[17] Suzuki Y (2010). Risk factors for severe hand foot and mouth disease. Pediatr Int.

[18] Pan J (2012). High risk factors for severe hand, foot and mouth disease: a multicenter retrospective survey in Anhui Province China, 2008–2009. Indian J Dermatol.

[19] Atkinson EJ (2012). Assessing fracture risk using gradient boosting machine (GBM) models. J Bone Miner Res.

[20] Chang LY (1999). Clinical features and risk factors of pulmonary oedema after enterovirus-71-related hand, foot, and mouth disease. Lancet.

[21] Huang CC (1999). Neurologic complications in children with enterovirus 71 infection. N Engl J Med.

[22] Chen SP (2010). Comparison of clinical features between coxsackievirus A2 and enterovirus 71 during the enterovirus outbreak in Taiwan, 2008: a children’s hospital experience. J Microbiol Immunol Infect.

[23] Ryu WS (2010). Clinical and etiological characteristics of enterovirus 71-related diseases during a recent 2-year period in Korea. J Clin Microbiol.

[24] Chen CS (2007). Retrograde axonal transport: a major transmission route of enterovirus 71 in mice. J Virol.

[25] Suzuki Y (2010). Risk factors for severe hand foot and mouth disease. Pediatr Int.

[26] Chang LY (1999). Clinical features and risk factors of pulmonary oedema after enterovirus-71-related hand, foot, and mouth disease. Lancet.

[27] Ooi MH (2009). Identification and validation of clinical predictors for the risk of neurological involvement in children with hand, foot, and mouth disease in Sarawak. Bmc Infect Dis.

[28] Li Y, Zhu R, Qian Y, Deng J (2012). The characteristics of blood glucose and WBC counts in peripheral blood of cases of hand foot and mouth disease in China: a systematic review. PLoS One.

[29] Shah VA (2003). Clinical characteristics of an outbreak of hand, foot and mouth disease in Singapore. Ann Acad Med Singapore.

[30] Chen F (2013). MRI characteristics of brainstem encephalitis in hand-foot-mouth disease induced by enterovirus type 71–will different MRI manifestations be helpful for prognosis?. Eur J Paediatr Neurol.

[31] Chen F (2014). MRI characteristics and follow-up findings in patients with neurological complications of enterovirus 71-related hand, foot, and mouth disease. Int J Clin Exp Med.

[32] Jang S (2012). Enterovirus 71-related encephalomyelitis: usual and unusual magnetic resonance imaging findings. Neuroradiology.

[33] Chen YC (2004). A murine oral enterovirus 71 infection model with central nervous system involvement. J Gen Virol.

[34] Wong KT (2008). The distribution of inflammation and virus in human enterovirus 71 encephalomyelitis suggests possible viral spread by neural pathways. J Neuropathol Exp Neurol.

[35] Nadel S (2013). Hand, foot, mouth, brainstem, and heart disease resulting from enterovirus 71. Crit Care Med.

