# Supplementary material for: Machine Learning Algorithms for Risk Prediction of Severe Hand-Foot-Mouth Disease in Children
Source: Sci Rep. 2017 Jul 14;7:5368. doi: 10.1038/s41598-017-05505-8 (PMC5511270; doi:10.1038/s41598-017-05505-8)
Supplement: Supplementary file 1 — supplementary data [file 41598_2017_5505_MOESM1_ESM.pdf]

Machine Learning Algorithms for Risk Prediction of Severe Hand-Foot-Mouth Disease in Children

Bin Zhang[1, 2†], Xiang Wan[3†], Fu-sheng Ouyang[4†], Yu-hao Dong[1†], De-hui Luo[5], Jing Liu [1, 2], Long Liang[1, 2], Wen-bo Chen[6], Xiao-ning Luo[1, 2], Xiao-kai Mo[1], Lu Zhang[1, 2], Wen-hui Huang[1], Shu-fang Pei[1, 2], , Bao-liang Guo[1, 2], Chang-hong Liang[1], Zhou-yang Lian[1*], Shui-xing Zhang[1*]

[1] Department of Radiology, Guangdong General Hospital/Guangdong Academy of Medical Sciences, Guangzhou, Guangdong, P.R. China

[2] Graduate College, Southern Medical University, Guangzhou, Guangdong, P.R. China

[3] Institute of Computational and Theoretical Study and Department of Computer Science, Hong Kong Baptist University, P.R. China

[4] Department of Radiology, The First People's Hospital of Shunde, Foshan, Guangdong, P.R. China

[5] Department of Mathematics, Hong Kong Baptist University, P.R. China

[6] Department of Radiology, Huizhou Municipal Central Hospital, Huizhou, Guangdong, P.R. China

†Bin Zhang, Xiang Wan, Fu-sheng Ouyang, and Yu-hao Dong contributed equally to this manuscript.

*Co-corresponding author: Shuixing Zhang and Zhouyang Lian

Department of Radiology, Guangdong General Hospital/Guangdong Academy of Medical Sciences, No. 106 Zhongshan Er Road, 510080 Guangzhou, Guangdong Prov., People's Republic of China

E-mail: shui7515@126.com

Tel: +86 20 83870125

Fax: +86 20 83870125

Supplementary Information

Statistical analysis

Uncovering the interactions of multiple attributes is a challenging problem. The conventional methods such as linear regression are limited both conceptually and statistically. First, the ability to detect multi-way interactions would be underpowered unless all multi-way interactions are pre-specified and explicitly formulated in the model. Second, when the number of possible configurations becomes very large while the sample size is limited, it is difficult to yield reliable statistical inferences from two-way or higher order interactions. In this study, we use the gradient boosting tree (GBT) approach to uncover interaction effects for the following reasons: (1) The GBT approach is a powerful machine learning technique combining the algorithms of decision trees and boosting, which can handle complex interaction effects that conventional approaches can not unravel; (2) It can handle different types of predictor variables and accommodate missing data; (3) There is no need for elimination of outliers and prior data transformation; (4) Given the non-linear nature of interaction patterns, GBT can provide robust non-linear solutions. Given these features, GBT is particularly suitable for handling interaction effects between factors in our study.

A Gradient Boosted Tree Approach

As aforementioned, non-linear based methods are preferred for solving the detection of multi-way interactions since interactions represent non-linear relationships among variables.

Tree-based models recently attract more attention in solving the identification of multi-way interactions. Unlike linear regression, there is no equation in tree-based models to express relationship between independent and dependent variables. Tree-based models partition the predictor space into rectangles, using a series of rules to identify regions having the most homogeneous responses to predictors. They then fit a constant to each region, with classification trees fitting the most probable class as the constant and regression trees fitting the mean response for observations in that region, assuming normally distributed errors. The hierarchical structure of a tree means that the response to one input variable depends on values of inputs higher in the tree, so interactions between predictors are automatically modelled. Furthermore, trees are insensitive to outliers, and can accommodate missing data in predictor variables by using surrogates.

Despite the above advantages, a single parsimonious tree has a number of limitations. It has difficulty in modelling smooth functions and the depth of the tree is not easy to decide. For a smaller tree, the effects of multi-way interactions could be under-estimated. For a larger tree, small changes in training data can result in very different tree structure. Gradient Boosting tree model solves this issue by combining the strengths of two algorithms: decision trees and boosting. Boosting is an adaptive method for combining many simple or weak models to give improved predictive performance. The gradient boosting tree approach is a powerful machine learning algorithm for regression, classification and ranking problems. The final tree model can be understood as a regression model with multiple trees adding together. It

produces a prediction model in the form of an ensemble of weak prediction models, typically decision trees.

In particular, boosting is a numerical optimization technique for minimizing the loss function by adding, at each step, a new tree that best reduces the loss function. It is easiest to explain in the least-squares regression setting, where the goal is to learn a model F that predicts values, minimizing the mean squared error to the true values y (averaged over some training sets). In boosting algorithm, the first tree is the one that, for the selected tree size, maximally reduces the loss function. For each following step, the focus is on the residuals, which is the variation in the response that is not so far explained by the model. The update at m step does not change. Instead, it improves on it by constructing a new tree that provide an estimator h on the residuals and then adding h into the current model to provide a better one. The final model is a linear combination of many trees (usually hundreds to thousands) that can be thought of as a regression model where each term is a tree.

Algorithm

To identify effective promotion configurations, we have an output variable y (i.e., sales, WOM impact, or conversion rate) and a vector of input variables x connected together via a joint probability distribution. Using a training set of known values of x and corresponding values of y, the goal is to find an approximation to a functionthat minimizes the expected value of some specified loss function:

The input of the model include: training set, a differentiable loss function, number of iterations M. The algorithm can be described as the following three steps:

(1) Initiate model with a constant value:.

(2) For to M:

Compute so-called pseudo-residuals: for i = 1,…,n.

Fit a base learner to pseudo-residuals, i.e., train it using the training set.

Compute multiplier by solving the following one-dimensional optimization problem: .

Update the model:.

(3) Output .

To summarize, the basic idea of gradient boosting tree approach is to fit an additive model F in a forward stage-wise manner. In each stage, it introduces a new regression tree h to compensate the shortcomings of existing model. The "Shortcomings" are identified by negative gradients. For any loss function, we can derive a gradient boosting algorithm. The tree-based methods provide a rigorous way to identify effective configurations that differ from the conventional correlational view (which implies linearity, additive effects, and unifinality), allowing complex causal relations to be explored in ways that generate new insight for configurational and strategy research (which stresses nonlinearity, synergistic effects, and equifinality). Recently, it has gained some popularity in the field of learning to rank.

The Procedure of Attributes and Interaction Selection

GBT is one of the most successful machine learning algorithms that has been successfully used in many areas. It provides high prediction accuracy and often outperforms many competing methods, such as linear regression/classification. It can

handle higher order interactions and produce highly complex functional forms. However, it is a non-parametric approach and the statistical inference could be an issue. For single variable, GBT will produce its relative importance (RI), which is based on the number of times a variable is selected for splitting, weighted by the squared improvement to the model as a result of each split, and averaged over all trees. The measures of relative importance of all variables are normalized and scaled to have a maximum value of 100. For the interaction, GBT will compute Friedman's H-statistic to assess the relative strength of interaction effects in non-linear models. H is on the scale of [0-1] with higher values indicating larger interaction effects. It is very difficult to select variables or interactions based on these measures without a well-defined threshold. In this work, we address this issue by conducting statistical inference on these measurements using permutation test.

A permutation test (also called a randomization test, re-randomization test, or an exact test) is a type of statistical significance test in which the distribution of the test statistic under the null hypothesis is obtained by calculating all possible values of the test statistic under rearrangements of the labels (values in our case) on the observed data points. The ranking of the real test statistic among the shuffled test statistics gives a p-value. Then we can use the standard p-value threshold 0.05 to select attributes and interactions. The outline of the permutation procedure is listed below:

1. Run GBT on the original data

2. Collect the measures of single variable V[1…n] and two-way interaction I[1…m]

3. T = 1000

4. Initialize matrix S1[1…n, 1…T], S2[1…m,1…T]

5. For t = 1 to 1000

 5.1 Permute the values of dependent variable and generate new data D’

 5.2 Run GBT on D’

 5.3 Collect the measures of single variable and save them to S1[1…n,t]

 5.4 Collect the measures of interactions and save them to S2 [1…m,t]

6. End For

7. The P value of ith variable is computed as the rank of V[i] in S1[i,1…1000].

8. The P values of jth interaction is computed as the rank of I[j] in S2[j,1…1000].

Statistical analysis were performed with R software (R Core Team. R: A language and environment for statistical computing. R Foundation for Statistical Computing, Vienna, Austria. URL http://www.R-project.org, 2016). The packages in R could be found in: https://cran.r-project.org/web/packages/gbm/gbm.pdf, including ‘gbm’, ‘glmnet’, ‘caret’, ‘gtools’, ‘ggplot2’, ‘gplots’, and ‘ROCR’. A p-value < 0.05 was deemed to be statistically significant based on two-sided tests.

Supplementary Figures

Figure 1. Boxplots of the test statistics for the individual predictors. Based on the definition of permutation test, for each variable, if more data points drop above the upper limit of the box, the p-value is more likely to be small than 0.05. It is evident that compare to other variables, body temperature, gender, age, rash or herpes and tachycardia have fewer (or nearly no) data laid above the upper limit of their boxes and hence their p-values are insignificant. Oppositely, increased WBC counts has the

maximum number of data drop above the upper limit of its box so it is the most influential predict factor of severe HFMD.

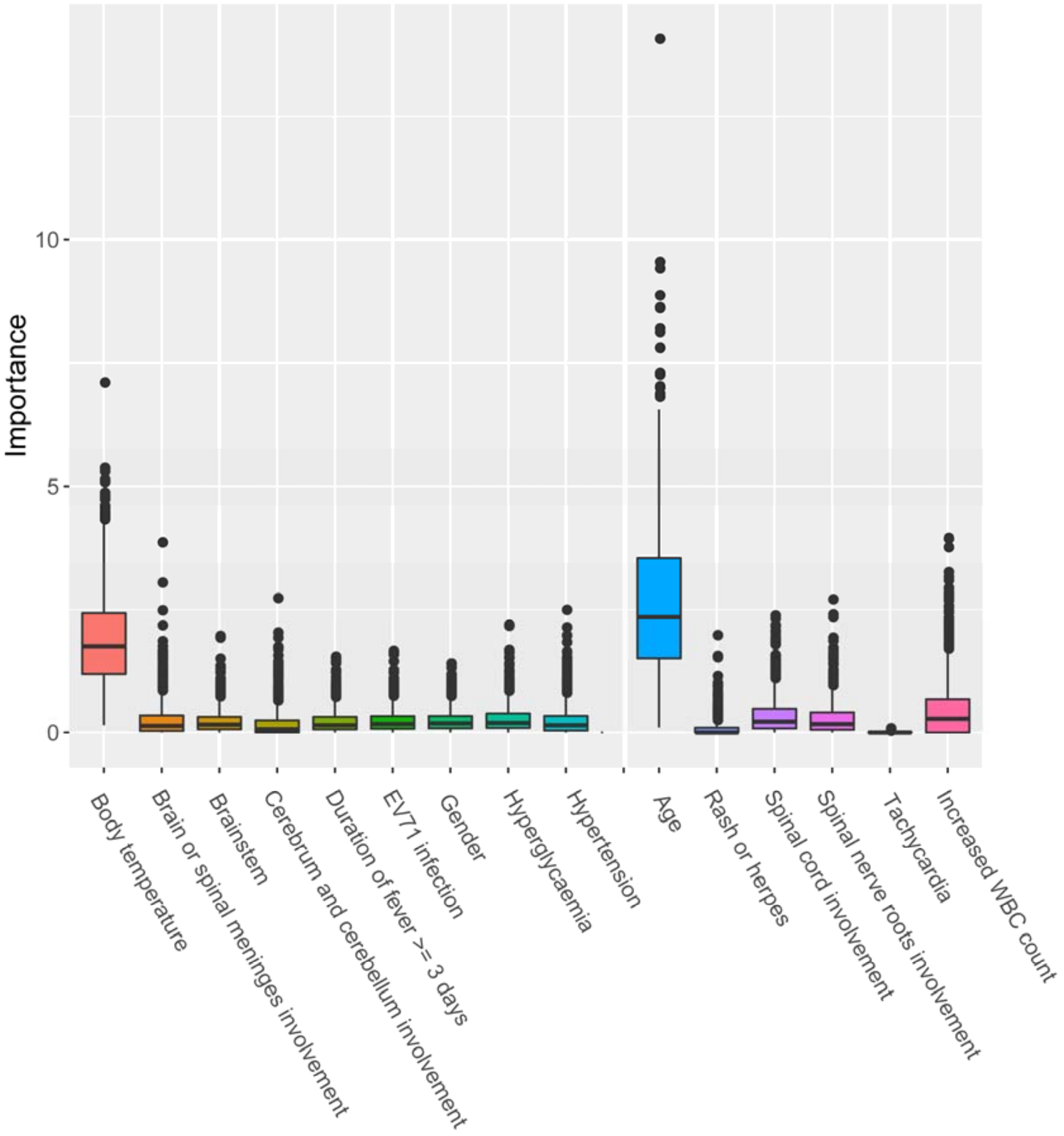

Figure 2. The performance of GBT model without using a balance approach

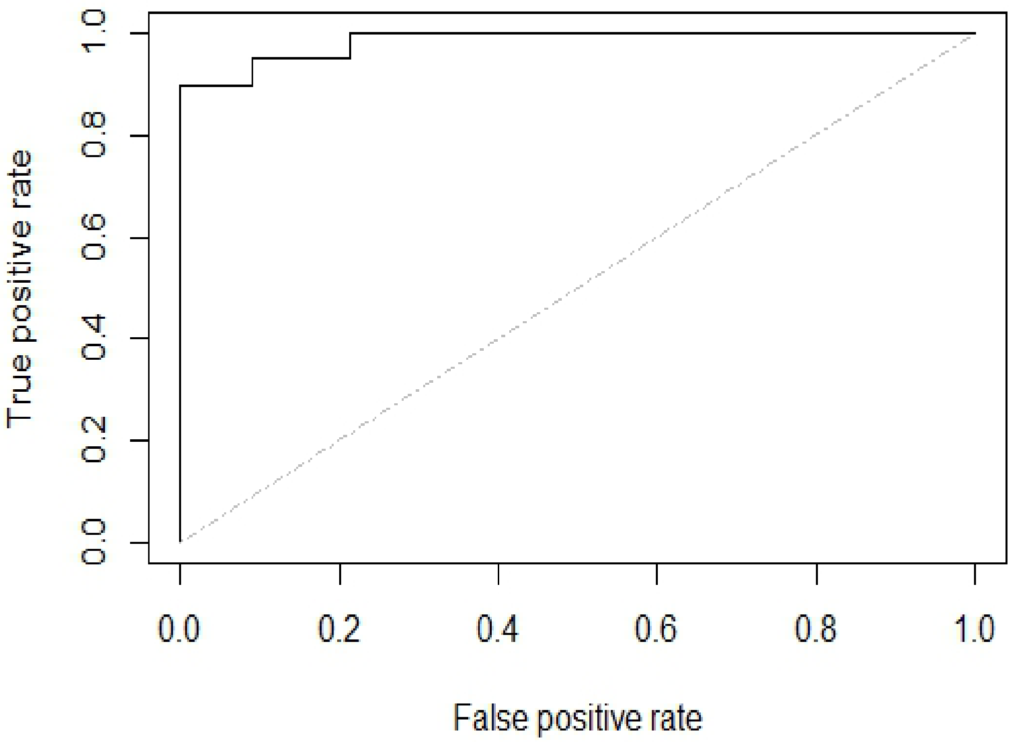

Figure 3. The performance of GBT model using a down-sampling procedure

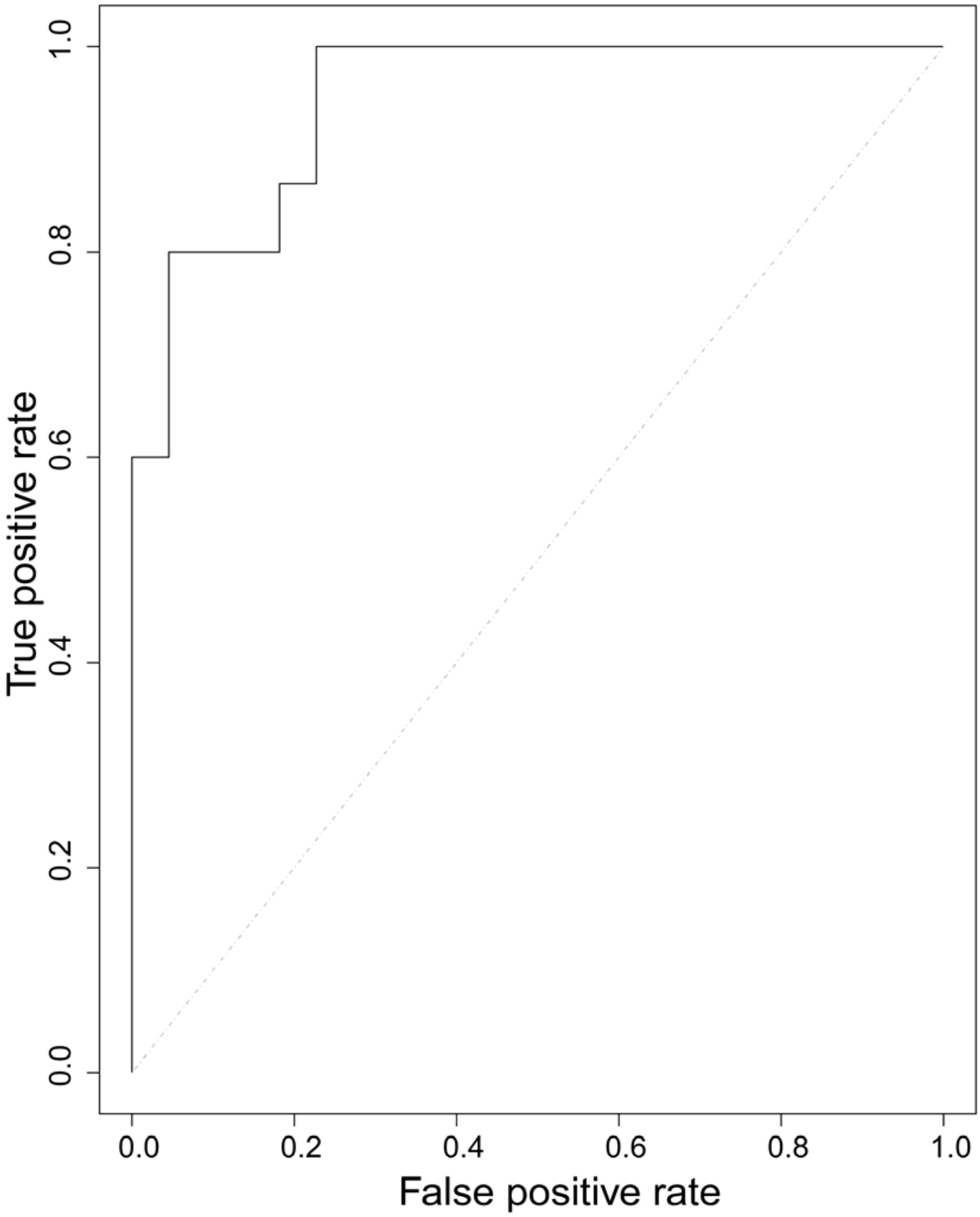

Figure 4. Boxplots of the H-statistics for interacting predictors. Similar to Figure 1, interactions between hyperglycemia and an increased WBC count, spinal nerve roots involvement and duration of fever⩾3 days relatively more significant as these two pairs of predictors have much more data points drop above the upper limit of their boxes. On the other hand, age and body temperature seem to have little interaction effect as there are only a few data are larger than its upper limit.

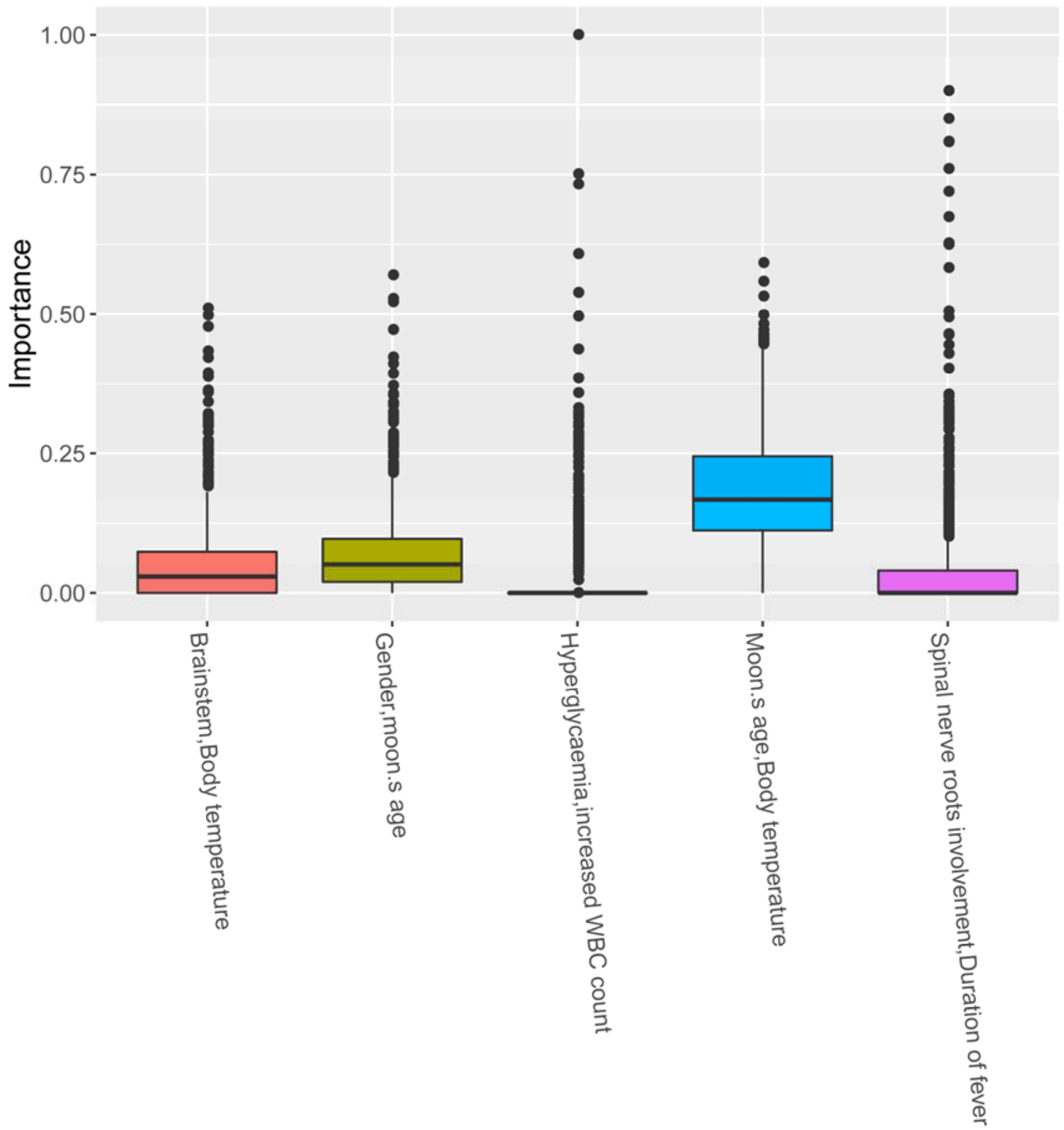

Importance
1.00
0.75
0.50
0.25
0.00
Brainstem,Body temperature
Gender,moon.s age
Hyperglycaemia,increased WBC count
Moon.s age,Body temperature
Spinal nerve roots involvement,Duration of fever
